# Working with Teenage Parents: Handbook of Theory and Practice for Practitioners Working with Pregnant Teenagers, Young Parents and Their Children

**DOI:** 10.1192/pb.bp.113.045401

**Published:** 2014-08

**Authors:** Sarah Jawad

*Working with Teenage Parents* is a textbook that aims to increase the clinical practitioner’s psychodynamic understanding of the ‘maturational processes’ that occur when adolescence and parenthood coincide. It works on the premise that in order to cultivate ‘developmental processes in the baby or toddler’, the young parent’s developmental needs also need to be met, which relies heavily on the skill of the practitioner.

The book is divided into five study modules: (1) interrelationships, (2) adolescents, (3) babies in teen families, (4) toddlers, and (5) families, groups and organisations. It enables a versatile approach to learning, accommodating both ‘self-reflective’ study and providing material, namely lecture handouts and interactive ‘role-play’ exercises, that can be delivered as part of formal group training. An interactive DVD is also included within the text to consolidate key learning points.

The intended aims and objectives for each module are clearly laid out and the often confusing, verbose explanations within the main text are concluded with key learning points. The glossary of terms and further reading recommendations within the training pack appendices are particularly useful.

The book’s aims to cater for every teaching eventuality did at times prove to be to its detriment due to difficulties in navigating between the appropriate sections and discarding instructions not relevant to the chosen mode of study. Despite encouragement to reflect on group exercise points, for those self-studying, like myself, this was not always practical or particularly fruitful; perhaps I would have had a more rewarding experience if I had studied with a group.

